# Supplementary material for: LncRNA OIP5-AS1 is overexpressed in undifferentiated oral tumors and integrated analysis identifies as a downstream effector of stemness-associated transcription factors
Source: Sci Rep. 2018 May 4;8:7018. doi: 10.1038/s41598-018-25451-3 (PMC5935738; doi:10.1038/s41598-018-25451-3)
Supplement: Supplementary file 1 — Supplementary File [file 41598_2018_25451_MOESM1_ESM.pdf]

LncRNA *OIP5-AS1* is overexpressed in undifferentiated oral tumors and integrated analysis identifies as a downstream effector of stemness-associated transcription factors.

Ganesan Arunkumar[1], Shankar Anand[1#], Partha Raksha[1#], Shankar Dhamodharan[1], Harikrishnan Prasanna Srinivasa Rao[2], Shanmugam Subbiah[2], Avaniyapuram Kannan Murugan[3], Arasambattu Kannan Munirajan[1].

[1]Department of Genetics, Dr. ALM PG Institute of Basic Medical Sciences, University of Madras, Taramani Campus, Chennai-600 113, India. [#]Indian Academy of Science Internship fellows.
[2]Center for Oncology, Royapettah Government Hospital & Kilpauk Medical College, Royapettah, Chennai-600 014, India.
[3]Department of Molecular Oncology, King Faisal Specialist Hospital and Research Center, Riyadh-11211, Saudi Arabia.

Correspondence to: A. K. Munirajan, **email:** akmunirajan@gmail.com; akmunirajan@unom.ac.in.

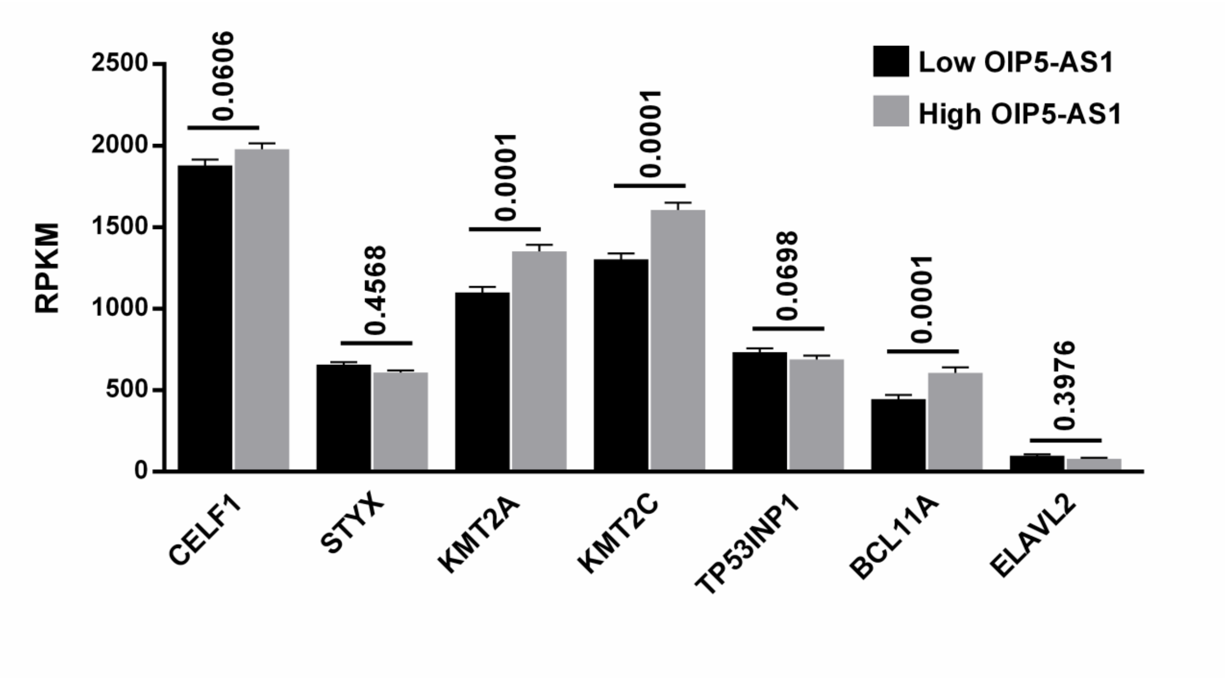

Supplement Figure S1: Expression of candidate genes with lncRNA *OIP5-AS1* levels. *KMT2A, KMT2C* and *BCL11A* were significantly upregulated in *OIP5-AS1* overexpressed HNSCC datasets from TCGA database. *CELF1* also showed overexpression with higher levels of *OIP5-AS1* in HNSCC.

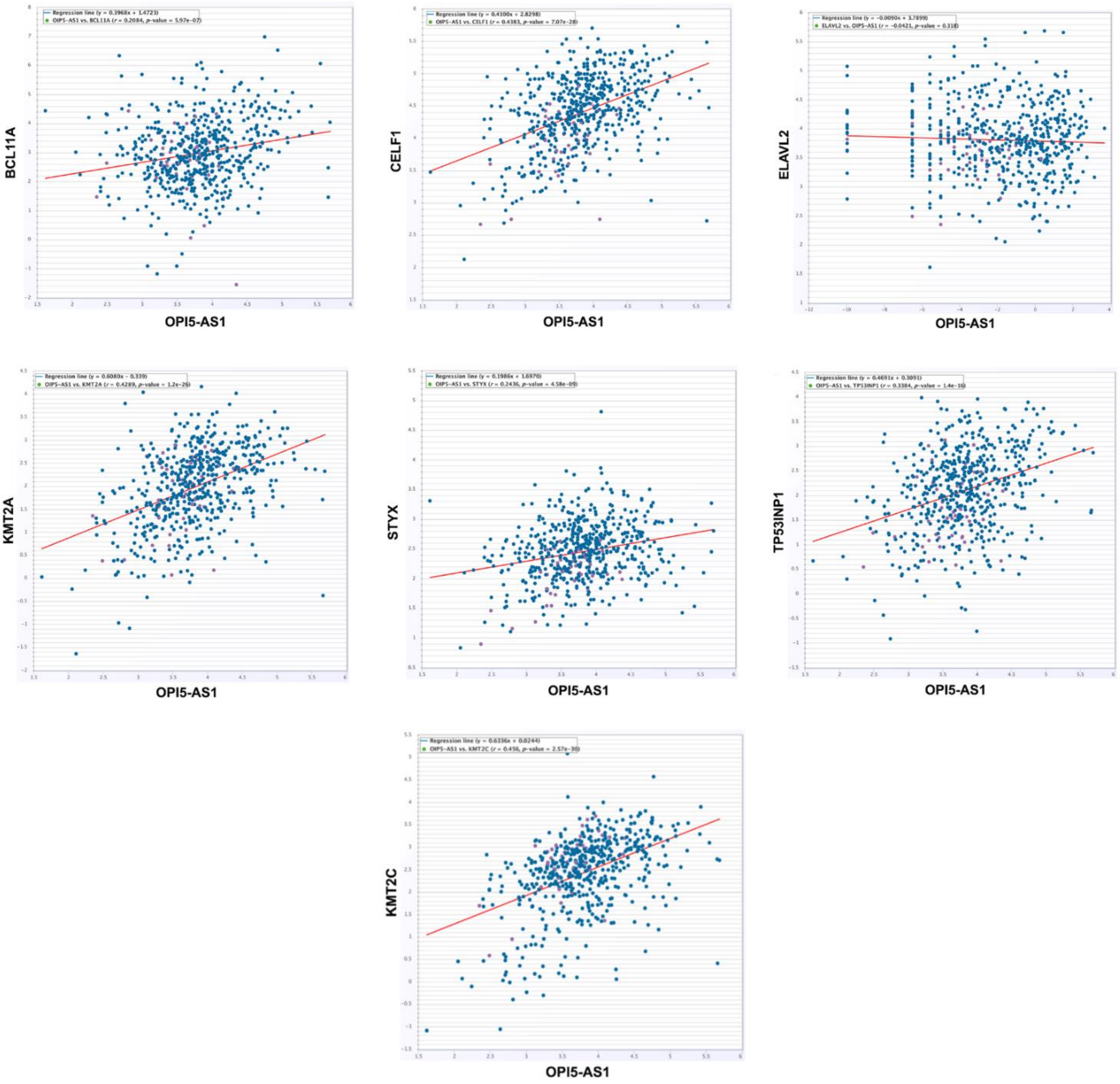

Supplement Figure S2: Correlation of candidate genes with *OIP5-AS1* expression. *CELF1*, *KMT2A* and *KMT2C* are having significant correlation with *OIP5-AS1* expression in head and neck cancer datasets from TCGA.

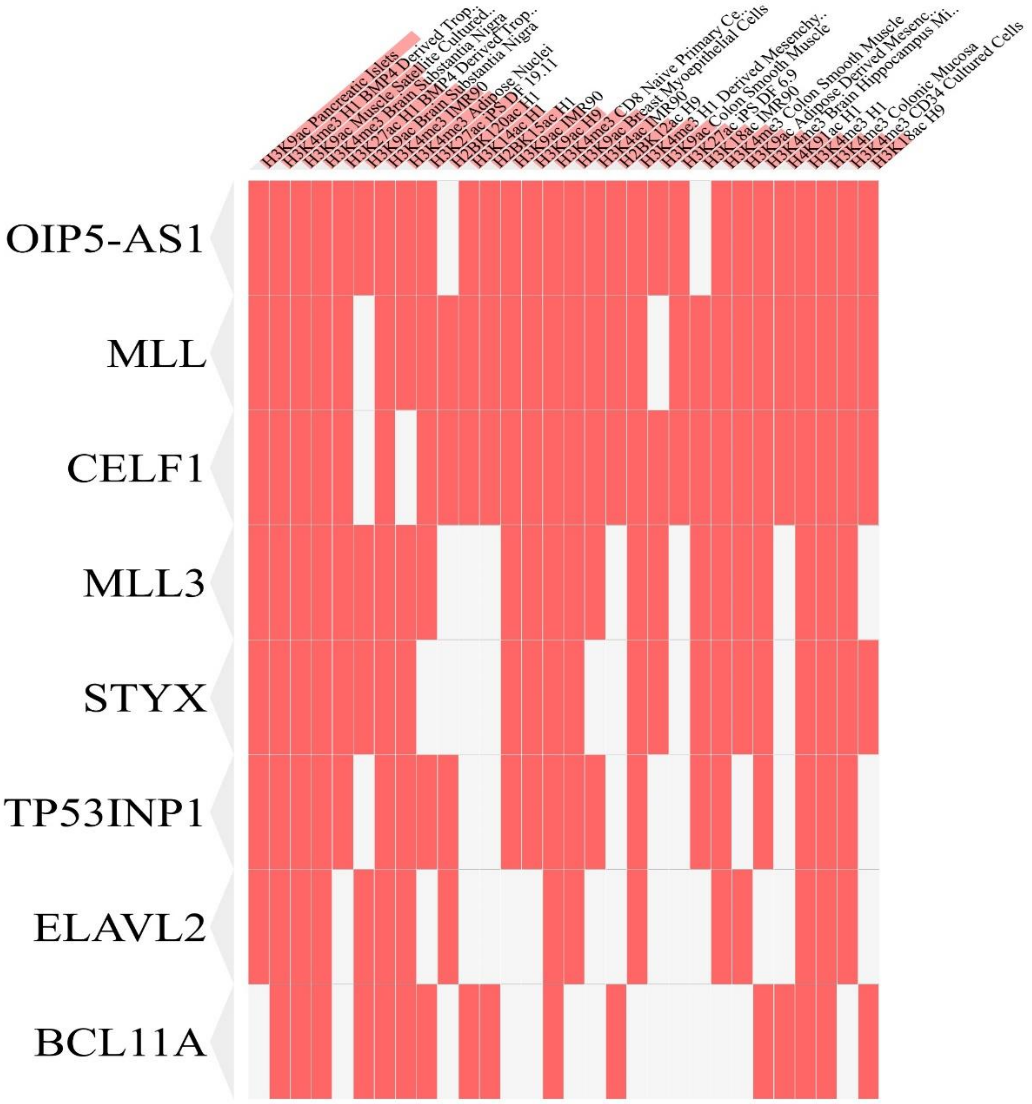

Supplement Figure S3: Chromatin modifications at candidate gene locus in human cell types. List of top 30 chromatin modifications at candidate gene locus in various human cell types by HM ChIP-seq from Roadmap Epigenomics Project. Along with lncRNA *OIP5-AS1* gene loci, *KMT2A*, *CELF1* and *KMT2C* are having significant active chromatin signature.

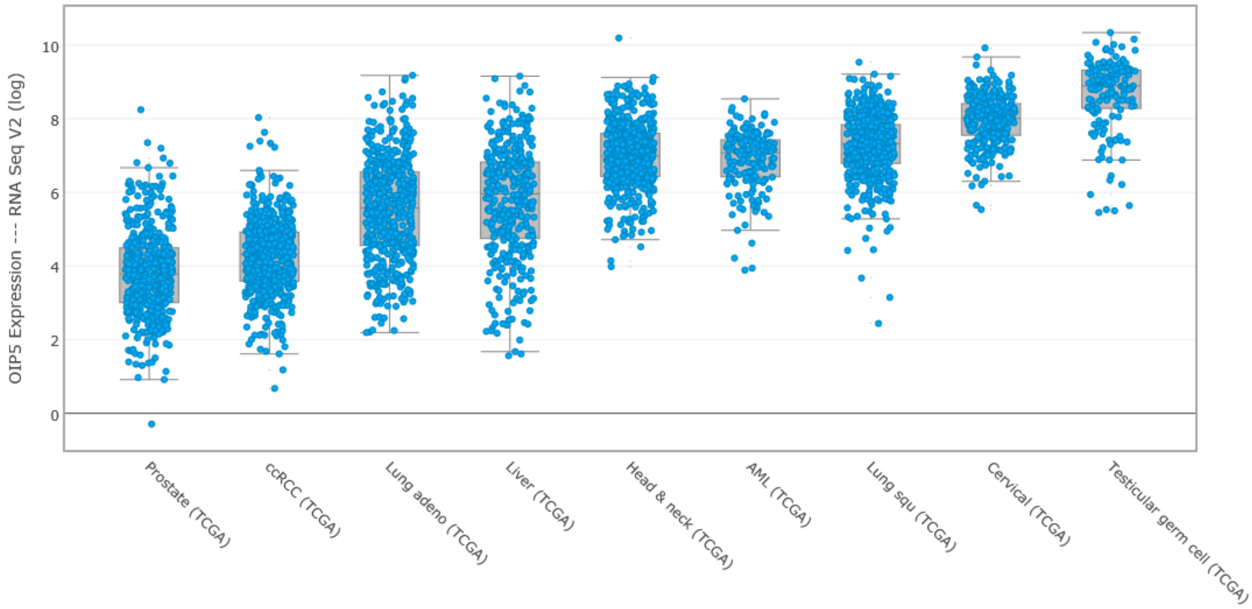

Supplement Figure S4: Expression of *OIP5* across different type of cancers from TCGA dataset. *OIP5* was significantly overexpressed in tumors of epithelial origin like *OIP5-AS1*. Testicular germ cell tumors expressed *OIP5* at very high level in comparison to all other tumors suggesting that *OIP5* also have a role in rapidly proliferating cells.

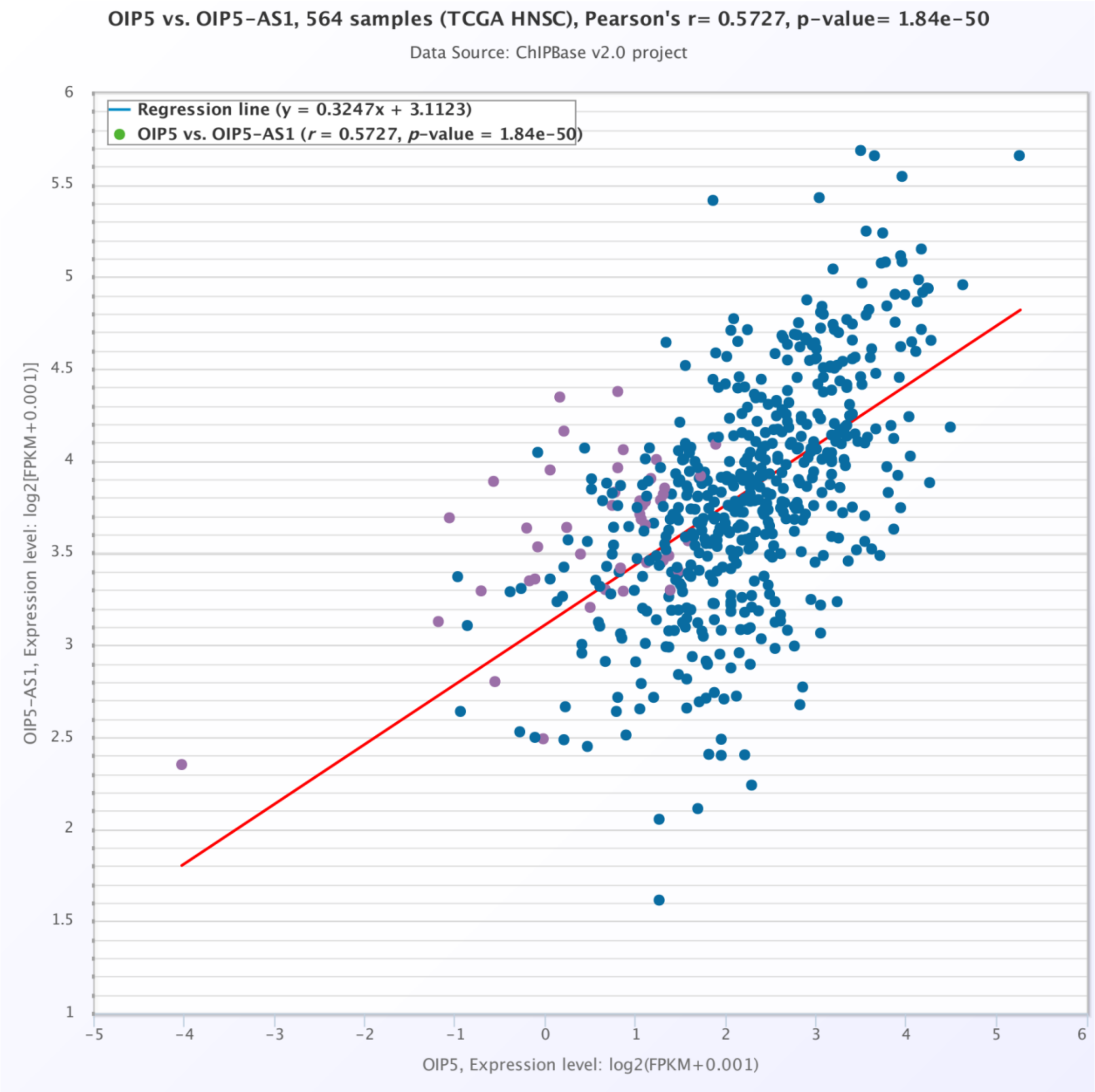

Supplement Figure S5: Correlation plot of *OIP5* with lncRNA *OIP5-AS1* expression. *OIP5* is the top significant co-expressed genes with LncRNA *OIP5-AS1* in HNSCC datasets of TCGA database (r = 0.5727).

Supplement Table S1: miRNAs binding sites in *NANOG* 3`-UTR

miRNAs for *NANOG*	Position in the UTR	seed match	context++ score	context++ score percentile	weighted context++ score	conserved branch length
hsa-miR-889-3p	15-21	7mer-m8	-0.15	96	-0.15	0.088
hsa-miR-6869-5p	20-26	7mer-1A	-0.13	92	-0.13	0
hsa-miR-4499	29-35	7mer-m8	-0.12	83	-0.12	0
hsa-miR-4494	30-36	7mer-m8	-0.16	86	-0.16	0
hsa-miR-198	33-40	8mer	-0.23	95	-0.23	0.01
hsa-miR-6733-3p	37-43	7mer-m8	-0.11	83	-0.11	0
hsa-miR-199a-5p	38-44	7mer-m8	-0.16	86	-0.16	0.055
hsa-miR-199b-5p	38-44	7mer-m8	-0.15	84	-0.15	0.055
hsa-miR-4676-5p	40-46	7mer-m8	-0.14	95	-0.14	0.01
hsa-miR-575	40-46	7mer-m8	-0.12	93	-0.12	0.01
hsa-miR-3157-5p	42-49	8mer	-0.35	98	-0.35	0
hsa-miR-4691-3p	42-48	7mer-1A	-0.08	77	-0.08	0
hsa-miR-541-5p	48-54	7mer-m8	-0.08	78	-0.08	0
hsa-miR-4533	50-56	7mer-m8	-0.21	93	-0.21	0
hsa-miR-6876-5p	51-57	7mer-m8	-0.17	91	-0.17	0
hsa-miR-4476	51-57	7mer-m8	-0.16	90	-0.16	0
hsa-miR-3916	53-59	7mer-m8	-0.07	68	-0.07	0
hsa-miR-3125	53-59	7mer-m8	-0.07	68	-0.07	0
hsa-miR-6859-5p	53-59	7mer-m8	-0.02	40	-0.02	0
hsa-miR-4728-5p	55-66	non-canonical	N/A	N/A	N/A	0
hsa-miR-4728-5p	55-66	non-canonical	N/A	N/A	N/A	0
hsa-miR-6731-5p	56-62	7mer-m8	-0.24	91	-0.24	0
hsa-miR-8085	56-62	7mer-m8	-0.23	90	-0.23	0
hsa-miR-3175	57-63	7mer-m8	-0.24	90	-0.24	0
hsa-miR-4779	61-67	7mer-m8	-0.24	93	-0.24	0
hsa-miR-765	63-69	7mer-m8	-0.28	93	-0.28	0.088
hsa-miR-7106-5p	64-71	8mer	-0.34	98	-0.34	0
hsa-miR-6799-5p	65-71	7mer-1A	-0.16	95	-0.16	0
hsa-miR-6780a-5p	65-71	7mer-m8	-0.29	94	-0.29	0
hsa-miR-6779-5p	65-71	7mer-m8	-0.3	94	-0.3	0
hsa-miR-30b-3p	65-71	7mer-m8	-0.26	93	-0.26	0
hsa-miR-3689b-3p	65-71	7mer-m8	-0.26	93	-0.26	0
hsa-miR-1273h-5p	65-71	7mer-m8	-0.27	93	-0.27	0
hsa-miR-3689c	65-71	7mer-m8	-0.26	93	-0.26	0
hsa-miR-3689a-3p	65-71	7mer-m8	-0.26	93	-0.26	0
hsa-miR-149-3p	65-71	7mer-1A	-0.23	91	-0.23	0
hsa-miR-4728-5p	65-71	7mer-1A	-0.23	90	-0.23	0
hsa-miR-6883-5p	65-71	7mer-1A	-0.19	88	-0.19	0
hsa-miR-6785-5p	65-71	7mer-1A	-0.19	87	-0.19	0
hsa-miR-6871-5p	66-72	7mer-m8	-0.24	92	-0.24	0
hsa-miR-6757-5p	70-76	7mer-m8	-0.26	93	-0.26	0
hsa-miR-6834-5p	72-78	7mer-m8	-0.26	96	-0.26	0

hsa-miR-6875-5p	72-78	7mer-1A	-0.18	89	-0.18	0
hsa-miR-6127	72-78	7mer-1A	-0.24	89	-0.24	0.01
hsa-miR-3126-5p	72-78	7mer-1A	-0.14	86	-0.14	0
hsa-miR-6129	72-78	7mer-1A	-0.21	86	-0.21	0.01
hsa-miR-6133	72-78	7mer-1A	-0.21	85	-0.21	0.01
hsa-miR-4510	72-78	7mer-1A	-0.21	85	-0.21	0.01
hsa-miR-4419a	72-78	7mer-1A	-0.2	83	-0.2	0.01
hsa-miR-6130	72-78	7mer-1A	-0.18	82	-0.18	0.01
hsa-miR-202-5p	77-83	7mer-m8	-0.2	92	-0.2	0.467
hsa-miR-4668-3p	81-87	7mer-m8	-0.06	94	-0.06	0
hsa-miR-548c-3p	82-88	7mer-m8	-0.02	84	-0.02	0.474
hsa-miR-29b-2-5p	97-103	7mer-m8	-0.05	74	-0.05	0
hsa-miR-589-3p	106-112	7mer-m8	-0.09	77	-0.09	0
hsa-miR-4261	113-119	7mer-1A	-0.15	86	-0.15	0
hsa-miR-3688-3p	114-120	7mer-m8	-0.02	29	-0.02	0
hsa-miR-490-5p	116-122	7mer-1A	-0.15	89	-0.15	0
hsa-miR-2681-3p	117-123	7mer-m8	-0.11	81	-0.11	0
hsa-miR-3945	122-128	7mer-1A	-0.16	75	-0.16	0
hsa-miR-6862-5p	122-128	7mer-1A	-0.13	61	-0.13	0
hsa-miR-4253	122-128	7mer-1A	-0.12	59	-0.12	0
hsa-miR-432-3p	126-132	7mer-m8	-0.23	90	-0.23	0
hsa-miR-1243	128-134	7mer-m8	-0.15	85	-0.15	0
hsa-miR-4439	131-137	7mer-1A	-0.13	87	-0.13	0.01
hsa-miR-3136-5p	131-137	7mer-1A	-0.17	86	-0.17	0.01
hsa-miR-943	131-137	7mer-1A	-0.12	82	-0.12	0.01
hsa-miR-5186	135-142	8mer	-0.29	98	-0.29	0
hsa-miR-6865-3p	145-151	7mer-m8	-0.19	89	-0.19	0
hsa-miR-3145-5p	150-156	7mer-1A	-0.21	94	-0.21	0.082
hsa-miR-508-5p	150-156	7mer-1A	-0.09	81	-0.09	0.082
hsa-miR-3920	168-174	7mer-m8	-0.02	26	-0.02	0
hsa-miR-6825-3p	171-177	7mer-1A	-0.17	80	-0.17	0
hsa-miR-3150b-5p	175-181	7mer-m8	-0.3	99	-0.3	0
hsa-miR-3150a-5p	175-181	7mer-m8	-0.3	99	-0.3	0
hsa-miR-4668-5p	183-194	non-canonical	N/A	N/A	N/A	0
hsa-miR-4668-5p	183-194	non-canonical	N/A	N/A	N/A	0
hsa-miR-4668-5p	189-200	non-canonical	N/A	N/A	N/A	0
hsa-miR-4668-5p	189-200	non-canonical	N/A	N/A	N/A	0
hsa-miR-3148	194-200	7mer-m8	-0.1	89	-0.1	0
hsa-miR-4496	196-202	7mer-1A	-0.04	90	-0.04	0
hsa-miR-6826-5p	199-205	7mer-m8	-0.23	94	-0.23	0
hsa-miR-323b-3p	201-207	7mer-1A	-0.04	66	-0.04	0.082
hsa-miR-665	211-217	7mer-1A	-0.24	95	-0.24	0.269
hsa-miR-3925-3p	213-219	7mer-1A	-0.1	84	-0.1	0
hsa-miR-766-3p	213-219	7mer-1A	-0.04	63	-0.04	0.01
hsa-miR-515-5p	214-220	7mer-1A	-0.13	86	-0.13	0.082
hsa-miR-519e-5p	214-220	7mer-1A	-0.12	84	-0.12	0.082

hsa-miR-629-3p	215-221	7mer-1A	-0.03	76	-0.03	0
hsa-miR-548l	222-228	7mer-m8	-0.09	92	-0.09	0.01
hsa-miR-548n	223-229	7mer-m8	-0.06	88	-0.06	0.01
hsa-miR-3613-3p	239-246	8mer	-0.16	96	-0.16	0
hsa-miR-607	242-248	7mer-1A	-0.01	40	-0.01	0
hsa-miR-1468-5p	246-252	7mer-m8	-0.21	67	-0.21	0
hsa-miR-4432	251-257	7mer-m8	-0.1	86	-0.1	0
hsa-miR-4696	253-259	7mer-m8	-0.2	92	-0.2	0
hsa-miR-451b	254-260	7mer-m8	-0.05	62	-0.05	0
hsa-miR-6501-3p	257-263	7mer-m8	-0.08	76	-0.08	0
hsa-miR-1273a	262-268	7mer-m8	-0.28	86	-0.28	0.01
hsa-miR-4649-5p	264-270	7mer-1A	-0.19	60	-0.19	0
hsa-miR-6729-5p	264-270	7mer-1A	-0.19	59	-0.19	0
hsa-miR-4486	265-271	7mer-m8	-0.14	93	-0.14	0
hsa-miR-939-3p	266-272	7mer-m8	-0.15	84	-0.15	0
hsa-miR-661	267-273	7mer-m8	-0.15	81	-0.15	0.01
hsa-miR-6849-3p	270-277	8mer	-0.18	92	-0.18	0
hsa-miR-6720-5p	271-277	7mer-m8	-0.17	87	-0.17	0
hsa-miR-6512-3p	271-277	7mer-m8	-0.15	84	-0.15	0
hsa-miR-766-3p	272-278	7mer-m8	-0.13	86	-0.13	0.01
hsa-miR-508-5p	273-279	7mer-m8	-0.07	78	-0.07	0
hsa-miR-7703	276-282	7mer-m8	-0.1	82	-0.1	0
hsa-miR-4793-3p	277-283	7mer-m8	-0.04	69	-0.04	0
hsa-miR-1273g-3p	280-286	7mer-m8	-0.15	84	-0.15	0
hsa-miR-4252	281-287	7mer-m8	-0.02	29	-0.02	0
hsa-miR-548ao-3p	289-295	7mer-m8	-0.03	37	-0.03	0
hsa-miR-6807-5p	295-301	7mer-m8	-0.02	32	-0.02	0
hsa-miR-6499-3p	300-306	7mer-1A	-0.06	77	-0.06	0
hsa-miR-455-3p.2	301-307	7mer-1A	-0.01	46	-0.01	0.018
hsa-miR-6516-5p	301-307	7mer-m8	-0.02	34	-0.02	0
hsa-miR-4327	303-309	7mer-m8	-0.05	46	-0.05	0
hsa-miR-139-3p	312-318	7mer-m8	-0.15	85	-0.15	0
hsa-miR-6746-5p	314-320	7mer-m8	-0.16	65	-0.16	0
hsa-miR-1247-3p	316-322	7mer-m8	-0.25	87	-0.25	0
hsa-miR-4768-3p	333-339	7mer-m8	-0.09	79	-0.09	0
hsa-miR-4722-5p	334-340	7mer-m8	-0.15	88	-0.15	0
hsa-miR-6808-5p	336-342	7mer-m8	-0.18	91	-0.18	0.01
hsa-miR-940	336-342	7mer-m8	-0.16	90	-0.16	0.01
hsa-miR-6893-5p	336-342	7mer-m8	-0.17	90	-0.17	0.01
hsa-miR-1827	337-344	8mer	-0.23	91	-0.23	0.01
hsa-miR-4649-3p	339-345	7mer-m8	-0.12	84	-0.12	0
hsa-miR-7160-5p	340-346	7mer-m8	-0.17	88	-0.17	0
hsa-miR-665	340-351	non-canonical	N/A	N/A	N/A	0
hsa-miR-665	340-351	non-canonical	N/A	N/A	N/A	0
hsa-miR-4478	342-348	7mer-m8	-0.03	80	-0.03	0
hsa-miR-3929	342-348	7mer-m8	-0.02	54	-0.02	0

hsa-miR-4419b	342-348	7mer-m8	-0.02	54	-0.02	0
hsa-miR-6884-5p	343-349	7mer-m8	-0.11	64	-0.11	0.735
hsa-miR-485-5p	343-349	7mer-m8	-0.09	63	-0.09	0.735
hsa-miR-6746-5p	347-353	7mer-1A	-0.24	85	-0.24	0
hsa-miR-6771-5p	347-353	7mer-m8	-0.22	73	-0.22	0
hsa-miR-423-3p	350-356	7mer-1A	-0.29	87	-0.29	0.012
hsa-miR-7977	357-363	7mer-m8	-0.05	68	-0.05	0
hsa-miR-5588-3p	359-366	8mer	-0.17	83	-0.17	0
hsa-miR-2114-5p	360-366	7mer-1A	-0.05	60	-0.05	0
hsa-miR-20b-3p	363-369	7mer-m8	-0.02	30	-0.02	0
hsa-miR-6720-3p	367-373	7mer-m8	-0.22	71	-0.22	0
hsa-miR-566	368-374	7mer-m8	-0.22	87	-0.22	0.01
hsa-miR-6499-5p	370-376	7mer-m8	-0.35	89	-0.35	0
hsa-miR-4767	371-377	7mer-m8	-0.31	88	-0.31	0
hsa-miR-663a	373-379	7mer-1A	-0.18	65	-0.18	0
hsa-miR-6787-5p	373-379	7mer-1A	-0.15	61	-0.15	0
hsa-miR-1908-5p	373-379	7mer-1A	-0.15	61	-0.15	0
hsa-miR-6815-5p	376-382	7mer-m8	-0.13	83	-0.13	0
hsa-miR-6865-5p	376-382	7mer-m8	-0.12	82	-0.12	0
hsa-miR-6134	377-383	7mer-m8	-0.1	60	-0.1	0
hsa-miR-381-5p	379-385	7mer-m8	-0.23	82	-0.23	0
hsa-miR-4649-5p	381-387	7mer-m8	-0.31	84	-0.31	0
hsa-miR-6729-5p	381-387	7mer-m8	-0.29	80	-0.29	0
hsa-miR-548c-3p	402-408	7mer-1A	-0.01	57	-0.01	0.01
hsa-miR-4684-5p	409-415	7mer-m8	-0.15	89	-0.15	0
hsa-miR-340-3p	412-418	7mer-m8	-0.31	82	-0.31	0
hsa-miR-6827-3p	412-418	7mer-m8	-0.26	75	-0.26	0
hsa-miR-6771-3p	417-423	7mer-m8	-0.16	89	-0.16	0
hsa-miR-128-3p	424-430	7mer-m8	-0.21	94	-0.21	0.018
hsa-miR-216a-3p	424-430	7mer-m8	-0.15	87	-0.15	0.018
hsa-miR-3681-3p	424-430	7mer-m8	-0.15	86	-0.15	0.018
hsa-miR-4794	432-438	7mer-m8	-0.16	91	-0.16	0
hsa-miR-664a-5p	432-438	7mer-m8	-0.14	85	-0.14	0
hsa-miR-8073	434-440	7mer-1A	-0.21	93	-0.21	0
hsa-miR-221-5p	434-440	7mer-1A	-0.21	92	-0.21	0
hsa-miR-4793-5p	436-442	7mer-m8	-0.13	85	-0.13	0
hsa-miR-6502-3p	440-446	7mer-m8	-0.09	85	-0.09	0
hsa-miR-4786-5p	441-447	7mer-m8	-0.15	82	-0.15	0
hsa-miR-4683	448-454	7mer-m8	-0.16	89	-0.16	0
hsa-miR-6888-5p	449-455	7mer-m8	-0.03	52	-0.03	0
hsa-miR-4768-3p	450-457	8mer	-0.2	96	-0.2	0
hsa-miR-4459	451-457	7mer-1A	-0.1	81	-0.1	0
hsa-miR-4433a-3p	451-457	7mer-1A	-0.05	77	-0.05	0.01
hsa-miR-3974	455-461	7mer-m8	-0.06	49	-0.06	0
hsa-miR-624-3p	457-463	7mer-m8	-0.09	79	-0.09	0.01
hsa-miR-4457	458-465	8mer	-0.25	96	-0.25	0

hsa-miR-513b-5p	459-465	7mer-m8	-0.07	78	-0.07	0.01
hsa-miR-125b-2-3p	459-465	7mer-1A	-0.03	62	-0.03	0
hsa-miR-3165	465-471	7mer-m8	-0.13	81	-0.13	0
hsa-miR-6745	466-472	7mer-m8	-0.28	94	-0.28	0
hsa-miR-363-5p	466-472	7mer-m8	-0.2	86	-0.2	0
hsa-miR-3656	468-474	7mer-m8	-0.24	79	-0.24	0
hsa-miR-6087	471-477	7mer-m8	-0.22	71	-0.22	0
hsa-miR-6089	477-483	7mer-m8	-0.16	93	-0.16	0
hsa-miR-4739	480-487	8mer	-0.3	96	-0.3	0
hsa-miR-1321	480-487	8mer	-0.27	95	-0.27	0
hsa-miR-4756-5p	480-487	8mer	-0.26	95	-0.26	0
hsa-miR-3162-5p	481-488	8mer	-0.25	90	-0.25	0
hsa-miR-6760-5p	481-487	7mer-1A	-0.14	83	-0.14	0
hsa-miR-1258	484-490	7mer-1A	-0.13	61	-0.13	0
hsa-miR-7977	491-497	7mer-m8	-0.14	88	-0.14	0
hsa-miR-605-5p	494-501	8mer	-0.16	92	-0.16	0
hsa-miR-3607-3p	498-504	7mer-m8	-0.02	46	-0.02	0
hsa-miR-4284	507-514	8mer	-0.04	79	-0.04	0
hsa-miR-24-3p	508-514	7mer-1A	-0.01	25	-0.01	0.018
hsa-miR-3178	516-522	7mer-m8	-0.31	93	-0.31	0
hsa-miR-6836-5p	519-525	7mer-m8	-0.19	85	-0.19	0.01
hsa-miR-6132	519-525	7mer-m8	-0.16	80	-0.16	0.01
hsa-miR-4763-3p	520-526	7mer-m8	-0.18	85	-0.18	0
hsa-miR-1207-5p	520-526	7mer-m8	-0.18	84	-0.18	0
hsa-miR-6808-5p	521-528	8mer	-0.23	95	-0.23	0.01
hsa-miR-940	521-528	8mer	-0.21	95	-0.21	0.01
hsa-miR-6893-5p	521-528	8mer	-0.23	95	-0.23	0.01
hsa-miR-6511a-5p	522-528	7mer-1A	-0.12	87	-0.12	0
hsa-miR-2682-5p	522-528	7mer-1A	-0.06	80	-0.06	0
hsa-miR-449c-5p	522-528	7mer-1A	-0.05	75	-0.05	0
hsa-miR-34b-5p	522-528	7mer-1A	-0.04	69	-0.04	0
hsa-miR-1910-3p	522-528	7mer-1A	-0.03	69	-0.03	0
hsa-miR-645	524-530	7mer-1A	-0.15	77	-0.15	0.01
hsa-miR-627-3p	528-535	8mer	-0.08	79	-0.08	0
hsa-miR-511-5p	531-537	7mer-m8	-0.1	90	-0.1	0.01
hsa-miR-6830-3p	531-537	7mer-1A	-0.03	59	-0.03	0
hsa-miR-3662	536-542	7mer-1A	-0.01	47	-0.01	0
hsa-miR-4477a	540-546	7mer-1A	-0.06	86	-0.06	0
hsa-miR-154-5p	543-549	7mer-m8	-0.23	94	-0.23	0.778
hsa-miR-575	562-568	7mer-1A	-0.12	93	-0.12	0
hsa-miR-4676-5p	562-568	7mer-1A	-0.1	92	-0.1	0
hsa-miR-6858-3p	562-568	7mer-1A	-0.1	85	-0.1	0
hsa-miR-3651	564-570	7mer-1A	-0.14	75	-0.14	0
hsa-miR-138-1-3p	574-580	7mer-1A	-0.07	86	-0.07	0
hsa-miR-7161-3p	578-584	7mer-1A	-0.13	87	-0.13	0
hsa-miR-3923	586-592	7mer-m8	-0.22	88	-0.22	0

hsa-miR-7844-5p	588-594	7mer-m8	-0.09	90	-0.09	0
hsa-miR-4744	600-606	7mer-m8	-0.1	81	-0.1	0
hsa-miR-652-5p	604-610	7mer-m8	-0.25	96	-0.25	0
hsa-miR-6771-3p	606-612	7mer-1A	-0.1	79	-0.1	0
hsa-miR-4799-5p	609-615	7mer-1A	-0.01	35	-0.01	0
hsa-miR-376a-5p	613-620	8mer	-0.23	97	-0.23	0.082
hsa-miR-4760-5p	614-620	7mer-m8	-0.03	61	-0.03	0
hsa-miR-8061	614-620	7mer-m8	-0.03	55	-0.03	0
hsa-miR-655-5p	618-625	8mer	-0.4	98	-0.4	0
hsa-miR-377-5p	619-625	7mer-1A	-0.17	86	-0.17	0.082
hsa-miR-6086	619-625	7mer-1A	-0.1	84	-0.1	0.082
hsa-miR-526b-5p	622-629	8mer	-0.37	98	-0.37	0.088
hsa-miR-643	636-642	7mer-m8	-0.23	94	-0.23	0.088
hsa-miR-186-3p	675-681	7mer-1A	-0.05	87	-0.05	0
hsa-miR-4733-5p	676-682	7mer-m8	-0.22	95	-0.22	0
hsa-miR-150-5p	682-688	7mer-m8	-0.18	96	-0.18	1.758
hsa-miR-6778-3p	684-690	7mer-m8	-0.37	98	-0.37	0
hsa-miR-7109-3p	686-692	7mer-m8	-0.29	97	-0.29	0
hsa-miR-1468-3p	691-703	non-canonical	N/A	N/A	N/A	0
hsa-miR-1468-3p	691-703	non-canonical	N/A	N/A	N/A	0
hsa-miR-6826-5p	710-716	7mer-1A	-0.12	81	-0.12	0
hsa-miR-4777-3p	714-721	8mer	-0.51	98	-0.51	0
hsa-miR-652-5p	722-728	7mer-1A	-0.22	94	-0.22	0
hsa-miR-6732-3p	722-728	7mer-1A	-0.18	88	-0.18	0
hsa-miR-4804-3p	725-731	7mer-m8	-0.23	96	-0.23	0
hsa-miR-567	735-741	7mer-m8	-0.19	91	-0.19	0.01
hsa-miR-4275	743-749	7mer-1A	-0.07	84	-0.07	0
hsa-miR-551b-5p	746-752	7mer-m8	-0.02	62	-0.02	0.082
hsa-miR-553	756-762	7mer-m8	-0.36	95	-0.36	0.01
hsa-miR-548p	772-779	8mer	-0.11	91	-0.11	0.01
hsa-miR-6824-5p	782-789	8mer	-0.54	99	-0.54	0
hsa-miR-6783-5p	782-788	7mer-1A	-0.21	90	-0.21	0
hsa-miR-4278	783-789	7mer-1A	-0.26	85	-0.26	0
hsa-miR-6789-5p	783-789	7mer-1A	-0.15	78	-0.15	0
hsa-miR-7-1-3p	789-795	7mer-m8	-0.02	72	-0.02	0
hsa-miR-7-2-3p	789-795	7mer-m8	-0.02	72	-0.02	0
hsa-miR-3065-5p	790-796	7mer-m8	-0.11	83	-0.11	0
hsa-miR-585-5p	799-806	8mer	-0.44	98	-0.44	0
hsa-miR-5682	800-806	7mer-1A	-0.1	79	-0.1	0
hsa-miR-524-5p	808-814	7mer-m8	-0.1	94	-0.1	0.082
hsa-miR-520d-5p	808-814	7mer-m8	-0.06	89	-0.06	0.082
hsa-miR-1283	809-816	8mer	-0.22	95	-0.22	0.01
hsa-miR-130b-5p	815-821	7mer-m8	-0.11	87	-0.11	0.082
hsa-miR-6821-3p	818-824	7mer-m8	-0.24	95	-0.24	0
hsa-miR-769-5p	820-826	7mer-m8	-0.22	94	-0.22	0
hsa-miR-545-3p	830-836	7mer-m8	-0.12	86	-0.12	0.088

hsa-miR-103a-3p	832-838	7mer-1A	-0.18	89	-0.18	0.986
hsa-miR-107	832-838	7mer-1A	-0.18	89	-0.18	0.986
hsa-miR-616-3p	843-849	7mer-1A	-0.13	80	-0.13	0.01
hsa-miR-6835-3p	857-863	7mer-1A	-0.04	81	-0.04	0
hsa-miR-4422	857-863	7mer-1A	-0.03	72	-0.03	0
hsa-miR-497-3p	866-872	7mer-1A	-0.09	89	-0.09	0
hsa-miR-302a-5p	868-874	7mer-m8	-0.13	96	-0.13	0.436
hsa-miR-203b-3p	873-879	7mer-1A	-0.17	92	-0.17	0
hsa-miR-7158-3p	873-879	7mer-1A	-0.16	92	-0.16	0
hsa-miR-664b-3p	877-884	8mer	-0.23	99	-0.23	0.088
hsa-miR-579-3p	877-884	8mer	-0.28	99	-0.28	0.088
hsa-miR-5696	878-884	7mer-1A	-0.02	61	-0.02	0
hsa-miR-664a-3p	879-885	7mer-m8	-0.08	88	-0.08	0.088
hsa-miR-1298-5p	882-888	7mer-1A	-0.12	91	-0.12	0.204
hsa-miR-3148	896-902	7mer-m8	-0.1	89	-0.1	0
hsa-miR-651-3p	898-904	7mer-m8	-0.11	90	-0.11	0
hsa-miR-4509	901-907	7mer-m8	-0.29	98	-0.29	0
hsa-miR-551b-5p	908-914	7mer-m8	-0.02	62	-0.02	0.082
hsa-miR-4668-3p	910-916	7mer-1A	-0.01	56	-0.01	0
hsa-miR-6823-5p	916-922	7mer-1A	-0.16	88	-0.16	0
hsa-miR-598-5p	925-931	7mer-1A	-0.32	95	-0.32	0
hsa-miR-653-5p	933-940	8mer	-0.25	97	-0.25	0.178
hsa-miR-494-3p	933-939	7mer-1A	-0.08	87	-0.08	0.456
hsa-miR-510-3p	934-940	7mer-m8	-0.16	97	-0.16	0
hsa-miR-4699-3p	943-950	8mer	-0.23	98	-0.23	0
hsa-miR-4999-5p	950-956	7mer-m8	-0.41	97	-0.41	0
hsa-miR-320e	953-959	7mer-1A	-0.23	94	-0.23	0
hsa-miR-4503	955-961	7mer-1A	-0.26	94	-0.26	0
hsa-miR-4307	959-965	7mer-1A	-0.17	96	0	0

Supplement Table S2: Functions of predicted miRNAs in various cancers.

Predicted miRNAs	Cancer types	Function/mechanism	Reference
hsa-miR-148a-3p	Gastric cancer	Tumor suppressor	PMID: 23456798
	Colorectal cancer	Poor overall survival	PMID: 23933284
	Pancreatic cancer	DNA hypermethylation	PMID: 20431052
	Hepatocellular carcinoma	Inhibits metastasis	PMID: 24798342
	Esophageal cancer	Recurrence/Survival	PMID: 20628822
	Breast cancer	Tumor suppressor	PMID: 23554686
	Ovarian cancer	Inhibits cell proliferation	PMID: 21971665
	Head and neck cancers	Inhibits metastasis	PMID: 18768788
hsa-miR-30a-5p	Lung adenocarcinoma	Inhibits tumor cell migration and invasion	PMID: 26837415
	Non-small cell lung cancer	Sensitizes radio-therapy	PMID: 28259977
	Ovarian cancer	Inhibits proliferation and invasion	PMID: 26675258
	Hepatocellular carcinoma	Inhibits tumor proliferation and promotes apoptosis	PMID: 26884832
	Head and neck cancers	Inhibits tumor cell progression	PMID: 26472042
hsa-miR-30b-5p	Gastric cancer	Tumor suppressor	PMID: 25170877
	Non-small cell lung cancer	Inhibits invasion and migration	PMID: 26388700
		Inhibits cell proliferation	PMID: 25249344
	Laryngeal carcinoma	Promotes p53 induced apoptosis	PMID: 25356506
	Breast cancer	Inhibits tumor cell growth and proliferation	PMID: 22384020
hsa-miR-338-3p	Glioblastoma	Inhibits tumor proliferation	PMID: 28493990
	Gastric cancer	Regulates EMT	PMID: 25945841
	Non-small cell lung cancer	Proliferation and apoptosis	PMID: 28428733
	Hepatocellular carcinoma	Inhibits cell growth and drug sensitization	PMID: 25531114
	Ovarian cancer	Inhibits cell proliferation and metabolism	PMID: 27508048
	Nasopharyngeal carcinoma	Inhibits tumor proliferation and migration	PMID: 26260688
hsa-miR-22-3p	Hepatocellular carcinoma	Inhibits cell proliferation	PMID: 27904693
		Post-transcriptional regulation	PMID: 28045918
hsa-miR-140-5p	Hepatocellular carcinoma	Cell growth and metastasis	PMID: 28383568
	Osteosarcoma	Inhibits tumor proliferation and signals autophagy	PMID: 27582507
	Cervical cancer	Growth arrest and inhibits metastasis	PMID: 27588393
	Breast cancer	Prevents tumor invasion	PMID: 25983620
	Hypopharyngeal carcinoma	Prevents tumor migration and invasion	PMID: 27033573
hsa-miR-148b-3p	Non-small cell lung cancer	Tumor suppressor	PMID: 25232379
		Radio-sensitization	PMID: 26759383
	Gastric cancer	Tumour cell metabolism	PMID: 28440026
	Breast cancer	Context-dependent tumor suppressive function	PMID: 25630670
hsa-miR-129-5p	Breast cancer	Regulates EMT	PMID: 26460733
	Lung cancer	Inhibits cell proliferation and tumor invasion	PMID: 28105223

	Gastric cancer	Reverses multi-drug resistance	PMID: 25344911
	Laryngeal carcinoma	Growth arrest and apoptosis	PMID: 24194897
	Hepatocellular carcinoma	Disease progression	PMID: 22536440
	Ovarian cancer	Inhibits cell proliferation and survival	PMID: 25895125
	Cervical cancer	anti-HPV activity	PMID: 24358111
hsa-miR-137	Neuroblastoma	Reverses drug resistance	PMID: 23934188
	Colorectal cancer	Inhibits cell proliferation	PMID: 27764771<br>PMID: 23275153
	Colorectal-Adeno carcinoma	Inhibits tumor progression	PMID: 28291253
	Ovarian cancer	Inhibit EMT and invasion	PMID: 27596137
	Breast cancer	Impairs tumor proliferative and migration	PMID: 22723937
	Renal cell carcinoma	Tumor suppressor	PMID: 27347205
	Lung cancer	Inhibits tumor growth and sensitizes chemosensitivity	PMID: 26989074
hsa-miR-30e-5p	Colorectal cancer	Inhibits invasion and metastasis	PMID: 28656629
	Breast cancer	Promotes proliferation, migration and invasion	PMID: 28288641
hsa-miR-363-3p	Head and neck cancers	Reduces cell migration	PMID: 26545583
	Lung adenocarcinoma	Inhibits tumor growth	PMID: 28423618
	Gastric cancer	Inhibits cell growth and migration	PMID: 26709677
	Papillary thyroid carcinoma	Inhibits tumor proliferation, migration and invasion	PMID: 28123856
	Gallbladder cancer	Controls tumor progression	PMID: 27420766
hsa-miR-424-5p	Neuroblastoma	Regulates ALK expression	PMID: 28455988
	Cervical cancer	Inhibits tumor cell growth	PMID: 28082020
	Gastric cancer	Promotes cell proliferation	PMID: 27655675
	Esophageal cancer	Prevents tumor invasion and metastasis	PMID: 27628042
	Non-small cell lung cancer	Inhibits proliferation, migration, and invasion	PMID: 27500472
	Oral cancer	Induce cell migration and invasion	PMID: 27038552
	Hepatocellular carcinoma	Inhibits cancer progression and EMT	PMID: 25175916
	Pancreatic cancer	Promotes tumor proliferation, migration and invasion	PMID: 23653113

Supplement Table S3: List of predicted target genes by *OIP5-AS1* sponged miRNAs

Supplement Table S4: List of RNA binding proteins and number of binding sites in predicted mRNAs

STYX		BCL11A		KMT2A		KMT2C		TP53INP1		CELF1		ELAVL2	
**RBPs**	**BS**	**RBPs**	**BS**	**RBPs**	**BS**	**RBPs**	**BS**	**RBPs**	**BS**	**RBPs**	**BS**	**RBPs**	**BS**
AGO1	1	AGO1	8	AGO1	4	AGO1	2	AGO2	43	AGO1	13	AGO2	6
AGO2	8	AGO2	42	AGO2	47	AGO2	37	ELAVL1	14	AGO2	55	ALKBH5	1
CPSF6	2	AGO3	3	AGO3	2	CPSF2	1	IGF2BP3	1	AGO3	3	C17orf85	1
CPSF7	3	AGO4	1	CPSF6	3	CPSF3	2	LIN28B	1	CPSF1	9	CPSF2	1
CSTF2	4	CAPRIN1	1	CPSF7	8	CPSF4	1	NOP56	1	CPSF3	1	CPSF6	2
CSTF2T	5	CPSF1	1	CSTF2	28	CPSF6	12	NOP58	1	CPSF6	23	CPSF7	2
ELAVL1	15	CPSF3	1	CSTF2T	6	CPSF7	30	TARDBP	1	CPSF7	17	CSTF2T	3
HNRNPC	5	CPSF6	5	DGCR8	1	CSTF2	81	TIAL1	2	CSTF2	43	ELAVL1	28
IGF2BP3	2	CPSF7	5	EIF4A3	11	CSTF2T	44	ZC3H7B	1	CSTF2T	26	FUS	22
MOV10	1	CSTF2T	9	ELAVL1	47	DGCR8	3			DGCR8	3	FXR2	1
PTBP1/PTBP2	1	ELAVL1	43	EWSR1	3	EIF4A3	39			EIF4A3	20	HNRNPA1	2
TARDBP	1	EWSR1	1	FIP1L1	1	ELAVL1	94			ELAVL1	140	HNRNPC	2
TIA1	1	FUS	18	FMR1	19	FIP1L1	6			EWSR1	1	HNRNPM	2
TIAL1	1	FXR1	1	FUS	12	FMR1	2			FIP1L1	15	IGF2BP2	1
YTHDF2	2	FXR2	1	FXR1	1	FUS	33			FMR1	1	LIN28B	1
		HNRNPA1	2	FXR2	2	HNRNPA1	9			FUS	21	MOV10	1
		HNRNPA2B1	1	HNRNPA1	5	HNRNPA2B1	2			HNRNPA1	1	NOP56	1
		HNRNPF	1	HNRNPC	22	HNRNPC	23			HNRNPA2B1	1	NUDT21	2
		HNRNPU	1	HNRNPM	3	HNRNPF	1			HNRNPC	170	TARDBP	1
		IGF2BP2	1	HNRNPU	2	HNRNPM	5			HNRNPH	9	TIAL1	1
		LIN28A	1	IGF2BP1	9	HNRNPU	3			HNRNPM	1	ZC3H7B	4
		LIN28B	3	IGF2BP2	2	IGF2BP1	3			HNRNPU	4		
		MOV10	2	IGF2BP3	1	IGF2BP3	1			IGF2BP1	4		
		NUDT21	4	LIN28A	4	LIN28A	1			IGF2BP2	1		
		PUM2	1	LIN28B	4	MOV10	1			LIN28A	3		
		TAF15	4	MOV10	8	NOP56	1			LIN28B	4		

		TARDBP	2	NUDT21	9	NUDT21	8			MOV10	15		
		ZC3H7B	8	PTBP1/PTBP2	12	PTBP1/PTBP2	16			NOP56	1		
				PUM2	1	RTCB	2			NUDT21	12		
				TAF15	2	TAF15	1			PTBP1/PTBP2	108		
				TARDBP	2	TARDBP	9			QKI	1		
				TIA1	2	TIA1	2			RTCB	2		
				TIAL1	2	TIAL1	8			TARDBP	13		
				YTHDF2	8	ZC3H7B	2			TIA1	2		
				ZC3H7B	3					TIAL1	18		
										YTHDF2	1		
										ZC3H7B	1		
**Total**													
15	52	28	171	35	296	34	485	9	65	37	763	21	85

RBP – RNA binding protein, BS – binding sites (n).

Supplement Table S5: List of histone modifications and TF bindings in candidate genes.

Supplement Table S6: List of stemness associated transcription factor binding motifs in *OIP5-AS1* gene

Regulator	Regulatory site	Binding locus	Binding site distance	Motif locus	Motif distance	Motif sequence
MYC	Upstream	chr15:41283601..41284186	-96	chr15:41283997..41284007	12	GCCACCTTGAG
MYC	Upstream	chr15:41283556..41284369	-27	chr15:41283767..41283777	-218	ACGGCGGAAAT
MYC	Upstream	chr15:41283570..41284236	-86	chr15:41283722..41283732	-263	GTCACGCGGCC
MYC	Upstream	chr15:41283548..41284261	-85	chr15:41283747..41283757	-238	TACCACGTGGT
MYC	Upstream	chr15:41283526..41284241	-106	chr15:41283747..41283757	-238	TACCACGTGGT
MYC	Upstream	chr15:41283091..41284467	-210	chr15:41283746..41283756	-239	TTACCACGTGG
MYC	Upstream	chr15:41283583..41283934	-231	chr15:41283747..41283757	-238	TACCACGTGGT
MYC	Upstream	chr15:41282946..41284373	-330	chr15:41283747..41283757	-238	TACCACGTGGT
NANOG	Upstream	chr15:41283621..41284153	-102	chr15:41283802..41283809	-184	ACAAAAGC
NANOG	Upstream	chr15:41283672..41284092	-107	chr15:41283802..41283809	-184	ACAAAAGC
NANOG	Upstream	chr15:41283792..41284034	-76	chr15:41283883..41283890	-103	GCATTCCA
KLF4	Upstream	chr15:41283769..41284136	-37	chr15:41283960..41283969	-25	GGGGCGGGGC
KLF4	Downstream	chr15:41284179..41284401	301	chr15:41284236..41284245	251	TGGGCGGGGG

Supplement Table S7. miRNAs interactions with *OIP5-AS1* associated lncRNA *TUG1*, *NEAT1*, *HOTAIR* and sense strand gene *OIP5*.

Supplement Table S8. List of Universal reverse transcription primers used for cDNA synthesis

Study type	Primer type	Sequence
LncRNA	Universal RT primer	5`CAGTGCAGGGTCCGAGGTACAGAGCCACCTGGGC AATTTTTTTTTTTTVN-3`
	Universal 2nd strand reverse primer	5`-CAGTGCAGGGTCCGAGGT-3`
miRNA	Stem loop RT primer – RNU44 specific	5`-GTCGTATCCAGTGCGTCGAGTGACACGAGAGCCA CCTGGGCAATTTGCACTGGATACGACAGTCAG- 3`
	Stem loop RT primer – miR-30b-5p specific	5`-GTCGTATCCAGTGCGTCGAGTGACACGAGAGCCA CCTGGGCAATTTGCACTGGATACGACAGCTGA- 3`
	Stem loop RT primer – miR-30a-5p specific	5`-TCGTATCCAGTGCGTCGAGTGACACGAGAGCCAC CTGGGCAATTTGCACTGGATACGACCTTCCA- 3`
	Stem loop RT primer – miR-338-3p specific	5`-TCGTATCCAGTGCGTCGAGTGACACGAGAGCCAC CTGGGCAATTTGCACTGGATACGACCAACAA- 3`
	Stem loop RT primer – miR-22-3p specific	5`-TCGTATCCAGTGCGTCGAGTGACACGAGAGCCAC CTGGGCAATTTGCACTGGATACGACACAGTT- 3`
	Stem loop RT primer – miR-140-5p specific	5`-TCGTATCCAGTGCGTCGAGTGACACGAGAGCCAC CTGGGCAATTTGCACTGGATACGACCTACCA- 3`
	Stem loop RT primer – miR-137 specific	5`-TCGTATCCAGTGCGTCGAGTGACACGAGAGCCAC CTGGGCAATTTGCACTGGATACGACCTACGC- 3`
	Stem loop RT primer – miR-148a-3p specific	5`-TCGTATCCAGTGCGTCGAGTGACACGAGAGCCAC CTGGGCAATTTGCACTGGATACGAC/ACAAAG- 3`
	Universal 2nd strand reverse primer	5`-TCGTATCCAGTGCGTCGAGT-3`
mRNA	Random hexamer primers	5`-NNNNNN-3`

3` Wobble bases: V- [A,C,G], N- [A,C,G,T]

Supplement Table S9. List of gene specific forward primers used for real time PCR experiments

ncRNAs	Forward primer sequences
GAPDH	5` - GAAGAGGGGAGGGGCCTAGG - 3`
OIP5-AS1	5'- GCTTCCAAATCAGCAGAGGACCAT - 3'
HOTAIR	5` - CTTGTGTAGGTTGTGTGTGTGTGGTGG - 3`
NEAT1	5` - TCTTCTTCCCCTTTACAGCACAAAT - 3`
TUG1	5' - GGCCGAGCGAACATGAAACTTTCAACT -3`
RNU44	5`- GCAAATGCTGACTGAACATGA - 3`
miR-30b-5p	5`- GCAGTGTAAACATCCTACACTCA - 3`
miR-30a-5p	5`- GCAGTGTAAACATCCTCGACT - 3`
miR-338-3p	5`- GCAGTCCAGCATCAGTGA - 3`
miR-22-3p	5`- AGCTGCCAGTTGAAGAAC - 3`
miR-140-5p	5`- CAGCAGTGGTTTTACCCTATG - 3`
miR-148a-3p	5` - CAGTCAGTGCACTACAGAACT - 3`
miR-137	5`- CGCAGTTATTGCTTAAGAATACG - 3`

Supplement Table S10. List of primers used for SYBR® Green gene expression assays

Gene	Forward	Reverse
GAPDH	5` - AGGGCTGCTTTTAACTCTGGT - 3`	5` - CCCCACTTGATTTGGAGGGA - 3`
CELF1	5` - CTGGACCACCCAGACCAACCA - 3`	5` - CATGCATCCCTGGGAGGACTTTCA - 3`
KMT2A	5` - CATCACCAGACCGACCTCCTCA - 3`	5` - GGACCGCTGGGGTGATAAGGAA -3`
KMT2C	5` - GGCTCATCACCGTTGTGTGGAGT - 3`	5` - GGGCTGTCGCACACTGCAC - 3`
